# Biomechanical Strategies for Minimizing Force Plate Targeting Effects during Running: Efficacy of Masked Force Plate Integration with Augmented Visual Feedback

**DOI:** 10.5114/jhk/212382

**Published:** 2025-11-20

**Authors:** Zifan Xia, Dong Sun, Yihan Qian, Yufan Xu, Chengyuan Zhu, Xuanzhen Cen, Yang Song, Liangliang Xiang, Monèm Jemni, Yaodong Gu

**Affiliations:** 1 Faculty of Sports Science, Ningbo University, Ningbo, China.; 2 Ningbo No. 2 Hospital, Zhejiang Engineering Research Center for New Technologies and Applications of Helium-Free Magnetic Resonance Imaging, Ningbo, China.; 3 Department of Biomedical Engineering, Faculty of Engineering, The Hong Kong Polytechnic University, Hong Kong, China.; 4 KTH MoveAbility Lab, Department of Engineering Mechanics, KTH Royal Institute of Technology, Stockholm, Sweden.; 5 Centre for Mental Health Research in Association with the University of Cambridge, Cambridge, United Kingdom.; 6 Research Institute of Sport Science, Hungarian University of Sports Science, Budapest, Hungary.

**Keywords:** gait analysis, targeting effect, step guidance strategy, joint kinematics, GRF, sEMG

## Abstract

This study aimed to investigate the targeting effect on the gait induced by the visual presence of a force plate during running and to develop a step guidance strategy (SGS) to minimize this effect and improve data acquisition success. Thirty-two healthy male participants were tested under three conditions: when the force plate was masked (MASK), when it was masked with the SGS implemented, and when it was visible (UNMASK). Kinematic, kinetic, and surface electromyography (sEMG) data were collected. The success rates for data acquisition were 30% for the MASK, 65.75% for the UNMASK, and 84.21% for the SGS condition. The UNMASK condition resulted in increased stride time, decreased stance time, and a lower coefficient of variation (CV) for the heel-to-force-plate distance. This condition also showed temporal variations in joint angles, an increased CV of the ankle joint angle waveform, significant alterations in ground reaction forces (GRF)—including greater peak braking force and impulses—and increased activation of the Vastus Medialis. The findings conclude that the visual presence of a force plate induces a targeting effect that undermines result reliability, and the proposed SGS effectively reduces this effect while significantly improving the success rate of data acquisition.

## Introduction

The human gait is one of the most extensively studied topics in the fields of biomechanics and rehabilitation science. Gait assessment can reveal many clinically meaningful phenomena, such as employing walking speed measurements to evaluate an individual's overall health status, differentiate among various pathological conditions, and assess the progression of diseases or changes in treatment efficacy (Cen et al., 2022, 2024; Fritz and Lusardi, 2009; Middleton et al., 2015; Miqueleiz et al., 2025).

Gait analysis must be based on the collection of natural, representative gait data that accurately reflect the subject's actual walking pattern, thereby ensuring that the experimental results possess high external validity, the ideal condition for gait experiments. When collecting kinetic data, subjects are typically required to place their feet fully on the force plate to achieve successful data acquisition (Gao et al., 2025; Song et al., 2025; Van der Meulen et al., 2024; Xu et al., 2025). The force plate is typically an embedded rectangular device with a limited area. During movement, to ensure that the foot is fully placed on the force plate for data collection, the subject is inevitably required to perform a stepping task. Under the influence of this task, the subject may adjust their movements based on the visual presence of the force plate, leading to unnatural gait modifications (Abendroth-Smith, 1996; Hirokawa, 1989; Rastegarpanah et al., 2018). This may result in gait data that do not accurately reflect the subject's natural movement state, potentially compromising the external validity of the experimental results (Imai et al., 2025). This phenomenon is known as the targeting effect of the force plate (Chen et al., 2025; Grabiner et al., 1995; Wearing et al., 2000).

The targeting effect has been considered in several studies. Abendroth-Smith (1996) and Rastegarpanah et al. (2018) confirmed the existence of the gait targeting effect by examining adjustments in spatiotemporal variables of the subjects’ gait. Hirokawa (1989) found that in constrained gait experiments, when subjects were required to step onto a marked square (similar to a force plate), gait timing was disturbed due to the appearance of a visual target. Research by Grabiner et al. (1995) and Wearing et al. (2000) indicated that visual guidance (i.e., actively aiming at the force plate) did not significantly affect ground reaction forces (GRFs). However, subsequent research by Wearing et al. (2003) demonstrated that visual guidance significantly altered the frequency components of GRFs in the medial-lateral and anterior-posterior directions. Furthermore, Sanderson et al. (1993) found that visual targets only modified the initial mechanical variables of the gait, while spatiotemporal variables remained stable. Verniba et al. (2015) discovered that in a young, healthy population, visual targets significantly reduced the variability in the distance between the foot and the target, although no differences were observed in the means or variability of spatiotemporal variables, kinematics, or kinetics. The existence of this targeting effect has raised concerns about whether gait data collected under these conditions are representative (Abendroth-Smith, 1996; Oggero et al., 1997). To overcome this targeting effect, researchers typically choose to mask the force plate to make it visually disappear (Bracht-Schweizer et al., 2017; Verniba et al., 2015). However, masking force plates, while preventing the targeting effect, reduces the success rate of data collection and adds considerable complications to studies involving special populations or requiring immediate post intervention measurements (Bracht-Schweizer et al., 2017).

Therefore, the aim of this study was to verify whether the targeting effect would occur during running when the force plate was visible and to propose a step guidance strategy (SGS) that was expected to eliminate the running targeting effect while increasing the probability of successful data collection. Our hypotheses were as follows: 1) the targeting effect exists when the force plate is visible to the subjects; specifically, running gait data collected under this condition may differ from these collected under a condition in which the force plate is not visible; 2) the step guidance strategy can effectively eliminate or reduce the targeting effect of the force plate; specifically, running gait data collected under this condition may show no significant differences compared to data collected when the force plate is not visible, or the differences may be reduced relative to those observed under the visible force plate condition; 3) the step guidance strategy will effectively increase the experimental success rate; that is, the number of attempts required to obtain enough usable data will be reduced under this condition.

## Methods

### Participants

A priori sample size calculation was performed using G*Power 3.1 software (Heinrich Heine University, Düsseldorf, Germany) (Faul et al., 2007). Based on the statistical framework of the paired sample *t*-test (i.e., the difference between two dependent means), the effect size (dz) was set at a medium level (Cohen’s *d* = 0.5) with target statistical power (1–β) of 0.75, indicating that a total sample size of at least 30 subjects was required. The inclusion criteria for participants were as follows: (1) age between 20 and 30 years; (2) no lower limb injury in the past six months; and (3) no lower limb abnormalities, gait abnormalities, or diseases. A total of 32 healthy male subjects with a rearfoot strike pattern were included in the study. All eligible participants were required to sign a written informed consent form approved by the institutional ethics committee of the Ningbo University prior to the experiment. The informed consent form detailed the purpose, procedures, potential risks, and rights of the participants, thereby ensuring that they voluntarily agreed to participate and were fully informed of all relevant details. This study was approved by the ethics committee of the Ningbo University, Ningbo, China (ethical approval number: TY2025028; approval date: 28 February 2025) and was conducted in strict accordance with the ethical principles of the Declaration of Helsinki.

### Experimental Procedure

**Figure 1:**
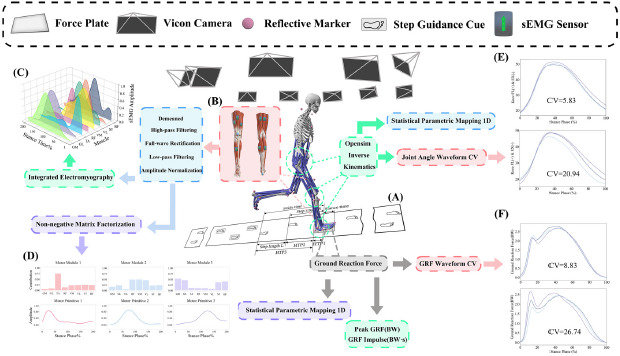
Flowchart of data acquisition and processing. (A) The runway and force plate (600 × 900 mm) were masked with paper. The force plate was visually occluded under MASK and SGS conditions; under the UNMASK condition, its outline was delineated using a marker pen. The gray, rounded rectangle located behind the force plate represents the step guidance cue (210 × 297 mm). (B) Placement of surface electromyography sensors. (C) Time-integrated surface electromyography (iEMG) from eight muscles. (D) Three muscle synergy modules extracted using non-negative matrix factorization (NMF): “Contribution” indicates the relative contribution of each muscle within a synergy, and “Amplitude” reflects the time-varying activation strength of each synergy during the task. (E & F) Example plots of the coefficient of variation (CV) for joint angles and ground reaction forces. *Note: GM = Medial Gastrocnemius; GL = Lateral Gastrocnemius; TA = Tibialis Anterior; RF = Rectus Femoris; VM = Vastus Medialis; VL = Vastus Lateralis; ST = Semitendinosus; BF = Biceps Femoris (Long Head);* EX = Extension; FL = Flexion. BW = Body Weight. Illustration is not to scale

Before the experiment began, 38 reflective markers were applied to the participants based on previously established protocols (Figure 1A) (Song et al., 2023, 2024, 2025). The entire procedure was carried out by the same operator. Following standard procedures, the skin was prepared for the placement of surface electromyography (sEMG) sensors (Wang et al., 2024), with eight surface EMG sensors being used (Figure 1B). The preparation and placement were performed by the same operator in accordance with the SENIAM guidelines. After completing the above procedures, participants performed a 10-min warm-up on a motorized treadmill at a self-selected speed.

**Figure 2:**
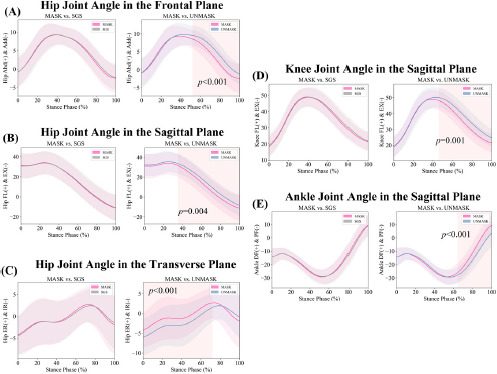
Temporal domain SPM1d comparison results of joint angles. (A) Hip joint angles in the coronal plane; (B) Hip joint angles in the sagittal plane; (C) Hip joint angles in the horizontal plane; (D) Knee joint angles in the sagittal plane; (E) Ankle joint angles in the sagittal plane. Note: Abd = Abduction; Add = Adduction; EX = Extension; FL = Flexion; IR = Internal Rotation; RE = External Rotation

**Figure 3:**
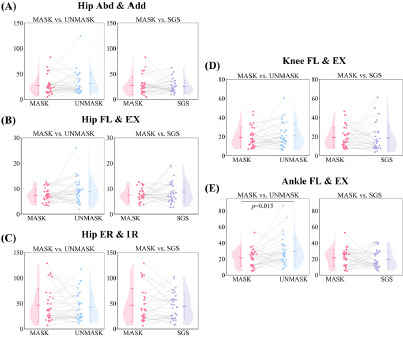
Paired sample *t*-test comparison results of the joint angle waveform CV; (A) hip joint angles in the coronal plane; (B) hip joint angles in the sagittal plane; (C) hip joint angles in the horizontal plane; (D) knee joint angles in the sagittal plane; (E) ankle joint angles in the sagittal plane. Note: Abd = Abduction; Add = Adduction; EX = Extension; FL = Flexion; IR = Internal Rotation; RE = External Rotation

**Figure 4:**
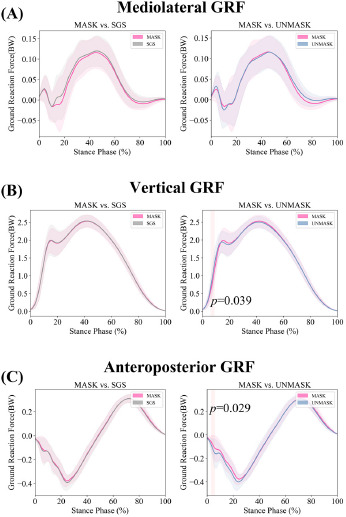
Temporal domain SPM1d comparison results of GRFs. Note: BW = Body Weight

**Figure 5:**
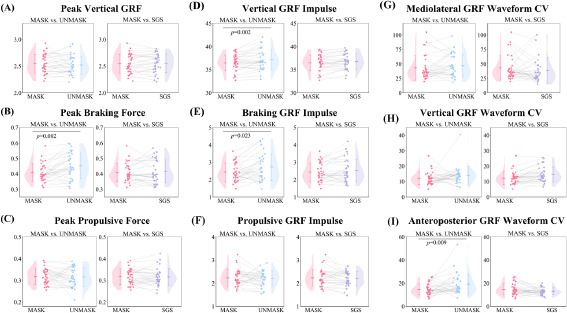
Paired sample *t*-test comparison results of GRF-related variables.

Participants then ran at a speed of 3.6 m/s ± 5% on an 18-m-long embedded force plate runway (Figure 1A), completing the running tasks under three different conditions (Table 1). Photocell timers were positioned at both ends of the force plate to measure the participants' speed. Participants first completed measurements under the MASK condition, in which both the force plate and the treadmill were masked and no visual cues were provided. This condition served as the gold standard in the current study. The typical step length and the stride step were calculated based on the coordinates of the heel reflective markers, using the average values obtained from three trials. Subsequently, the step guidance cues were positioned as follows: the first cue was placed at a distance equivalent to three times the participant’s typical step length posterior to the center of the force plate. The second cue was positioned one step length behind the first cue, and the third cue was placed one step length behind the second. The mediolateral distance between the three cues corresponded to the participant’s typical step width. Together, these three cues formed the Step Guidance Strategy (SGS) configuration (Figure 1A), which was then used for data collection under the SGS condition. During the SGS trials, participants were not required to step precisely on the cues. Instead, they were instructed that the cues served merely as visual references, and that approximate foot placement on or near the cue area was sufficient. Finally, measurements were taken under the UNMASK condition, where participants could see the force plate. However, under the UNMASK condition, verbal instructions were provided to prevent participants from directly looking at the force plate or intentionally adjusting their steps. A successful trial was defined as the complete placement of the participant's dominant foot on the force plate. Each participant was required to successfully complete three data collection trials under each condition. The required number of data collections and the criteria for a successful trial were not disclosed to the participants. To avoid residual effects from the previous experimental condition, a 20-min washout period was implemented between each condition.

**Table 1:** Data collection conditions and descriptions.

Conditions	Description
MASK	Data were collected on a visually masked runway
SGS	The force plate was visually masked, and a step guidance strategy was implemented in front of the plate (Figure 1A)
UNMASK	The force plate was visually present (outlined on the masked runway)

Kinematic data were captured using the Vicon three-dimensional motion capture system (Vicon Metrics Ltd., Oxford, United Kingdom) equipped with 10 cameras at a frequency of 200 Hz (Zhu et al., 2024). Ground reaction forces (GRFs) were collected using the Kistler three-dimensional force plate (Kistler, Winterthur, Switzerland) at a frequency of 2000 Hz (Chang et al., 2024). Surface electromyography (sEMG) signals were synchronized and recorded using the Delsys surface electromyograph (Delsys, Boston, USA) at a sampling frequency of 2000 Hz (Liu and Fernandez, 2024).

### Data Process and Analysis

The data were first processed using Vicon Nexus 2.15 software (Vicon Motion Systems, Oxford, United Kingdom). GRFs and kinematic signals were low-pass filtered with fourth-order, zero-lag Butterworth filters at cut-off frequencies of 50 Hz and 12 Hz, respectively. Gait events were defined by a vertical GRF threshold exceeding 10 N. For gait events not involving contact with the force plate, the method proposed by Zeni et al. (2008) was used. Specifically, this method detects foot-strike events by identifying the transition of the anterior-posterior velocity vector of the heel marker from positive to negative, and detects toe-off events by identifying the transition of the anterior-posterior velocity vector of the toe marker from negative to positive. A standardized workflow using OpenSim 4.3 (SimTK, Stanford University, Stanford, USA) was then performed as previously established (Delp et al., 2007). Joint angles were computed using inverse kinematics. Joint kinematics and GRF data were time-normalized to 101 data points using interpolation in Python 3.13 (Python Software Foundation, Wilmington, USA) for subsequent analyses. The raw sEMG signals were first demeaned to eliminate DC offsets, followed by a fourth-order Butterworth high-pass filter with a cut-off frequency of 50 Hz to remove low-frequency noise. The signals were then full-wave rectified. Subsequently, a fourth-order Butterworth low-pass filter with a cut-off frequency of 12 Hz was used to smooth the signals and extract the signal envelope. To facilitate comparisons across different experimental conditions, the sEMG amplitude was normalized using each participant's individual global maximum value. Finally, the time series of the signals was normalized to 200 data points (Santuz, 2022). All sEMG signal processing procedures were performed using Python.

The spatiotemporal gait variables of interest included the coefficient of variation (CV) of the heel-to-force plate (HTP) distance over three consecutive steps, left step length, right step length, stride time, and stance time (Figure 1A). Kinematic analyses included the temporal differences in three-dimensional hip joint angles and the sagittal plane angles of the knee and the ankle during the stance phase, as well as the waveform CV of joint angles (O’Dwyer et al., 2009). Kinetic variables included the vertical GRF peak, the vertical GRF impulse, braking and propulsive GRF peaks, and braking and propulsive GRF impulses. The waveform CV was also calculated for GRF curves in the mediolateral, vertical, and anteroposterior directions. sEMG signals were analyzed using non-negative matrix factorization (NMF) to examine differences in muscle synergy activation patterns (Fan et al., 2024; Wang et al., 2024). The number of muscle synergies to be extracted was determined based on achieving a variance accounted for (VAF) greater than 90% (Wang et al., 2024). NMF was conducted using RStudio (Integrated Development for R. RStudio, PBC, Wilmington, USA). Additionally, the integrated EMG (iEMG) of each muscle during the stance phase was computed. Furthermore, the success rate of data acquisition under each experimental condition was included in the analysis.

### Statistical Analysis

For all data, the Shapiro-Wilk test was first performed on the pairwise differences to assess normality. If the data followed a normal distribution, paired sample *t*-tests were conducted for discrete variables, and one-dimensional Statistical Parametric Mapping (SPM1d) was applied for paired comparisons of continuous variables. If the data did not follow a normal distribution, the Wilcoxon signed-rank test was used for discrete variables, and the non-parametric paired *t*-test from the SPM1d package was applied to continuous variables. To test Hypothesis I, comparisons were performed between the MASK and UNMASK conditions. To test Hypothesis II, comparisons were performed between the MASK and SGS conditions. The significance level for all statistical analyses was set at 0.05. Tests on discrete variables were performed using IBM SPSS Statistics 27 (IBM Corp., Armonk, NY, USA), while SPM1d analyses were conducted using the SPM1d package in Python.

## Results

### Data Collection Success Rate

Under the MASK condition, participants required a total of 320 attempts to achieve 96 successful data collections, yielding a success rate of 30%. Under the SGS condition, the success rate was approximately 84.21%, with 114 attempts required to complete the data collection. Under the UNMASK condition, the success rate was approximately 65.75%, with 146 attempts completed.

### Spatiotemporal Measures

Compared to the MASK condition, the UNMASK condition exhibited a significantly longer stride time (0.75 ± 0.05 s vs. 0.74 ± 0.04 s, *p* = 0.017, *d* = 0.446) and a significantly shorter stance time (0.22 ± 0.02 s vs. 0.24 ± 0.02 s, *p* < 0.001, *d* = 3.976). The HTP3 CV of the UNMASK condition was significantly lower compared to the MASK condition (42.50 ± 36.49% vs. 68.67 ± 44.74%, *p* = 0.012, *d* = 0.473), while no significant differences were observed in the remaining variables. In the comparison between the MASK and SGS conditions, the HTP1 CV was significantly lower under the SGS condition (6.45 ± 4.37% vs. 3.01 ± 2.02%, *p* < 0.001, *d* = 0.736). Additionally, the HTP2 CV under the SGS condition was significantly lower than under the MASK condition (13.57 ± 8.89% vs. 7.63 ± 4.85%, *p* = 0.002, *d* = 0.611), with no significant differences observed for the other variables (Table 2).

**Table 2:** Paired sample *t*-test comparison results of spatiotemporal measures.

Comparison	Variables	Mean (SD)		*t*	*p*	Cohen’s *d* (95% CI)
		MASK	UNMASK			
MASKvs.UNMASK	Stride time (s)	0.74 (0.04)	0.75 (0.05)	2.523	**0.017**	0.446 (0.079, 0.806)
	Stance time (s)	0.24 (0.02)	0.22 (0.02)	22.492	**< 0.001**	3.976 (2.928, 5.015)
	Step length-L (mm)	1225.70 (170.56)	1236.16 (201.95)	0.241	0.811	0.043 (–0.304, 0.389)
	Step length-R (mm)	1292.55 (163.61)	1324.34 (165.78)	0.864	0.394	0.153 (–0.197, 0.500)
	HTP1 CV (%)	6.45 (4.37)	6.68 (5.45)	0.181	0.857	0.032 (–0.315, 0.378)
	HTP2 CV (%)	13.57 (8.89)	14.55 (15.46)	0.287	0.776	0.051 (–0.296, 0.397)
	HTP3 CV (%)	68.67 (44.74)	42.50 (36.49)	2.677	**0.012**	0.473 (0.104, 0.836)
	Variables	Mean (SD)		*t*	*p*	Cohen’s *d* (95% CI)
		MASK	SGS			
MASKvs.SGS	Stride time (s)	0.74 (0.04)	0.75 (0.04)	1.995	0.055	0.353 (–0.007, 0.707)
	Stance time (s)	0.24 (0.02)	0.24 (0.02)	1.82	0.078	0.322 (-0.036, 0.675)
	Step length-L (mm)	1225.70 (170.56)	1236.89 (90.62)	0.401	0.691	0.071 (–0.277, 0.417)
	Step length-R (mm)	1292.55 (163.61)	1266.02 (89.17)	0.974	0.338	0.172 (−0.178, 0.520)
	HTP1 CV (%)	6.45 (4.37)	3.01 (2.02)	4.161	**< 0.001**	0.736 (0.339, 1.122)
	HTP2 CV (%)	13.57 (8.89)	7.63 (4.85)	3.457	**0.002**	0.611 (0.229, 0.985)
	HTP3 CV (%)	68.67 (44.74)	50.27 (51.30)	1.987	0.056	0.351 (−0.009, 0.706)

Note: Boldface indicates a significant difference (p < 0.05)

### Kinematic Variables

Significant differences were observed between the MASK and UNMASK conditions across various phases of the stance period in the selected three joints with five degrees of freedom. Specifically, in the sagittal plane, the hip joint flexion angle was significantly greater under the UNMASK condition from 34% to 100% of the stance phase. In the coronal plane, the hip abduction angle was significantly greater under the UNMASK condition from 52% to 100% of the stance phase. Additionally, in the transverse plane, the UNMASK condition showed a significantly greater hip internal rotation angle from 0% to 72% of the stance phase. At the knee joint, the flexion angle was significantly greater under the UNMASK condition from 46% to 100% of the stance phase. At the ankle joint, greater plantarflexion and reduced dorsiflexion angles were observed under the UNMASK condition from 64% to 100% of the stance phase. No significant differences were observed between the MASK and SGS conditions.

The comparison revealed that under the UNMASK condition, the ankle joint angle waveform CV was significantly higher than under the MASK condition (29.58 ± 17.42% vs. 21.50 ± 10.61%, *p* = 0.015, *d* = 0.455), with no significant differences observed in the other joints. Furthermore, no significant differences were observed in the comparison between the MASK and SGS conditions.

### Kinetic Variables

During the 6–9% phase of the stance, the vertical GRF under the UNMASK condition was higher compared to the MASK condition. Additionally, during the 3–6% phase of the stance, the anteroposterior GRF under the UNMASK condition was significantly lower than in the MASK condition. No significant differences were observed in the comparisons between the MASK and SGS conditions.

Compared to the MASK condition, under the UNMASK condition, the measured peak braking force (0.45 ± 0.08 BW vs. 0.41 ± 0.06 BW, *p* = 0.002, *d* = 0.598), the vertical GRF impulse (37.15 ± 2.13 BW•s vs. 36.48 ± 1.71 BW•s, *p* = 0.002, *d* = 0.584), the braking GRF impulse (2.74 ± 0.79 BW•s vs. 2.43 ± 0.53 BW•s, *p* = 0.023, *d* = 0.424), and the anteroposterior GRF waveform CV (19.21 ± 8.79% vs. 14.62 ± 4.85%, *p* = 0.009, *d* = 0.492) were significantly greater, while no significant differences were observed in other variables. No significant differences were observed in the comparisons between the MASK and SGS conditions.

### Electromyographic Activity

From the motor primitive in Figure 1D, it can be observed that Synergy 1 approximately represented the loading response phase (5–15% of the stance phase), Synergy 2 approximately represented the mid-stance phase (15–40% of the stance phase), and Synergy 3 approximately represented the propulsion phase (40–60% of the stance phase).

In Synergy 3, the weight of VM in the synergy under the UNMASK condition was significantly higher than that under the MASK condition (0.13 ± 0.09 vs. 0.07 ± 0.07, *p* = 0.032, *d* = 0.478). Furthermore, no significant differences were observed in the other comparisons (Table 5).

**Table 3:** Paired sample *t*-test comparison results of muscle weights in Synergy 1.

Comparison	Variables	Mean (SD)		*t*	*p*	Cohen’s *d* (95% CI)
		MASK	UNMASK			
MASKvs.UNMASK	GM	0.06 (0.07)	0.06 (0.04)	0.216	0.831	0.045 (−0.364, 0.453)
	GL	0.11 (0.12)	0.10 (0.11)	0.521	0.608	0.109 (−0.303, 0.517)
	TA	0.76 (0.13)	0.75 (0.10)	0.141	0.889	0.029 (−0.380, 0.438)
	RF	0.18 (0.12)	0.12 (0.10)	1.836	0.08	0.383 (−0.045, 0.803)
	VM	0.20 (0.12)	0.20 (0.10)	0.02	0.984	0.004 (–0.405, 0.413)
	VL	0.24 (0.12)	0.24 (0.13)	0.003	0.998	0.001 (−0.408, 0.409)
	ST	0.21 (0.19)	0.22 (0.12)	0.359	0.723	0.075 (−0.335, 0.483)
	BF	0.23 (0.13)	0.27 (0.16)	1.103	0.282	0.230 (–0.187, 0.642)
	Variables	Mean (SD)		*t*	*p*	Cohen’s *d* (95% CI)
		MASK	SGS			
MASKvs.SGS	GM	0.06 (0.07)	0.06 (0.05)	0.026	0.980	0.005 (−0.403, 0.414)
	GL	0.11 (0.12)	0.09 (0.08)	0.858	0.4	0.179 (−0.235, 0.589)
	TA	0.76 (0.13)	0.79 (0.12)	0.942	0.357	0.196 (–0.219, 0.607)
	RF	0.18 (0.12)	0.15 (0.09)	0.737	0.469	0.154 (−0.259, 0.563)
	VM	0.20 (0.12)	0.21 (0.11)	0.196	0.846	0.041 (–0.368, 0.449)
	VL	0.24 (0.12)	0.22 (0.12)	0.657	0.518	0.137 (−0.275, 0.546)
	ST	0.21 (0.19)	0.23 (0.19)	0.657	0.518	0.137 (–0.275, 0.546)
	BF	0.23 (0.13)	0.22 (0.12)	0.244	0.809	0.051 (−0.359, 0.459)

Note: GM = Medial Gastrocnemius; GL = Lateral Gastrocnemius; TA = Tibialis Anterior; RF = Rectus Femoris; VM = Vastus Medialis; VL = Vastus Lateralis; ST = Semitendinosus; BF = Biceps Femoris (Long Head)

**Table 4:** Paired sample *t*-test comparison results of muscle weights in Synergy 2.

Comparison	Variables	Mean (SD)		*t*	*p*	Cohen’s *d* (95% CI)
		MASK	UNMASK			
MASKvs.UNMASK	GM	0.19 (0.12)	0.12 (0.11)	1.927	0.067	0.402 (−0.028, 0.823)
	GL	0.23 (0.13)	0.23 (0.15)	0.088	0.93	0.018 (−0.391, 0.427)
	TA	0.08 (0.14)	0.08 (0.13)	0.141	0.889	0.029 (−0.380, 0.438)
	RF	0.44 (0.13)	0.51 (0.07)	2.013	0.057	0.420 (–0.012, 0.842)
	VM	0.45 (0.13)	0.46 (0.09)	0.457	0.652	0.095 (− 0.315, 0.504)
	VL	0.46 (0.13)	0.47 (0.11)	0.414	0.683	0.086 (–0.324, 0.495)
	ST	0.20 (0.13)	0.22 (0.14)	0.608	0.549	0.127 (–0.285, 0.536)
	BF	0.20 (0.14)	0.20 (0.12)	0.016	0.988	0.003 (−0.405, 0.412)
	Variables	Mean (SD)		*t*	*p*	Cohen’s *d* (95% CI)
		MASK	SGS			
MASKvs.SGS	GM	0.19 (0.12)	0.17 (0.10)	0.671	0.509	0.140 (−0.272, 0.549)
	GL	0.23 (0.13)	0.24 (0.14)	0.221	0.827	0.046 (–0.363, 0.454)
	TA	0.08 (0.14)	0.04 (0.09)	1.094	0.286	0.228 (−0.189, 0.640)
	RF	0.44 (0.13)	0.49 (0.06)	1.793	0.087	0.374 (–0.053, 0.793)
	VM	0.45 (0.13)	0.49 (0.06)	1.299	0.208	0.271 (–0.149, 0.684)
	VL	0.46 (0.13)	0.46 (0.11)	0.189	0.852	0.039 (–0.370, 0.448)
	ST	0.20 (0.13)	0.21 (0.12)	0.508	0.617	0.106 (–0.305, 0.515))
	BF	0.20 (0.14)	0.21 (0.16)	0.79	0.938	0.016 (–0.392, 0.425)

Note: GM = Medial Gastrocnemius; GL = Lateral Gastrocnemius; TA = Tibialis Anterior; RF = Rectus Femoris; VM = Vastus Medialis; VL = Vastus Lateralis; ST = Semitendinosus; BF = Biceps Femoris (Long Head)

**Table 5:** Paired sample *t*-test comparison results of muscle weights in Synergy 3.

Comparison	Muscles	Mean (SD)		*t*	*p*	Cohen’s *d* (95% CI)
		MASK	UNMASK			
MASKvs.UNMASK	GM	0.52 (0.13)	0.54 (0.15)	0.599	0.555	0.125 (–0.287, 0.534)
	GL	0.51 (0.16)	0.44 (0.18)	1.135	0.269	0.237 (−0.181, 0.649)
	TA	0.12 (0.16)	0.11 (0.17)	0.259	0.798	0.054 (−0.356, 0.462)
	RF	0.09 (0.06)	0.09 (0.06)	0.247	0.807	0.052 (−0.358, 0.460)
	VM	0.07 (0.07)	0.13 (0.09)	2.294	**0.032**	0.478 (0.041, 0.906)
	VL	0.07 (0.07)	0.08 (0.08)	0.572	0.573	0.119 (–0.292, 0.528)
	ST	0.40 (0.17)	0.33 (0.17)	1.195	0.245	0.249 (−0.169, 0.662)
	BF	0.31 (0.17)	0.40 (0.16)	2.194	0.039	0.457 (0.023, 0.883)
	**Muscles**	**Mean (SD)**		***t***	***p***	**Cohen’s *d* (95% CI)**
		**MASK**	**SGS**			
MASKvs.SGS	GM	0.52 (0.13)	0.53 (0.11)	0.305	0.763	0.064 (–0.346, 0.472)
	GL	0.51 (0.16)	0.47 (0.17)	0.874	0.391	0.182 (−0.232, 0.592)
	TA	0.12 (0.16)	0.10 (0.18)	0.671	0.509	0.140 (−0.272, 0.549)
	RF	0.09 (0.06)	0.11 (0.08)	1.109	0.279	0.231 (–0.186, 0.643)
	VM	0.07 (0.07)	0.10 (0.07)	1.381	0.181	0.288 (–0.132, 0.702)
	VL	0.07 (0.07)	0.07 (0.06)	0.082	0.935	0.017 (–0.392, 0.426)
	ST	0.40 (0.17)	0.34 (0.15)	1.089	0.288	0.227 (−0.190, 0.639)
	BF	0.31 (0.17)	0.40 (0.17)	1.727	0.098	0.360 (–0.066, 0.778)

Note: GM = Medial Gastrocnemius; GL = Lateral Gastrocnemius; TA = Tibialis Anterior; RF = Rectus Femoris; VM = Vastus Medialis; VL = Vastus Lateralis; ST = Semitendinosus; BF = Biceps Femoris (Long Head). Boldface indicates a significant difference (p < 0.05)

The iEMG of the VM under the UNMASK condition was significantly higher than that under the MASK condition (76.64 ± 15.07 %peak⋅%Stance vs. 72.19 ± 15.47 %peak⋅%Stance, *p* = 0.021, *d* = 0.429). Furthermore, no significant differences were observed in the other comparisons (Table 6).

**Table 6:** Paired sample *t*-test comparison results of iEMG (%peak⋅%Stance).

Comparison	Muscles	Mean (SD)		*t*	*p*	Cohen’s *d* (95% CI)
		MASK	UNMASK			
MASKvs.UNMASK	GM	85.76 (17.31)	85.67 (12.07)	0.026	0.98	0.005 (−0.342, 0.351)
	GL	89.14 (19.93)	84.91 (18.81)	1.197	0.24	0.212 (−0.140, 0.560)
	TA	71.25 (20.85)	69.21 (22.55)	0.45	0.656	0.080 (−0.268, 0.426)
	RF	76.74 (13.69)	78.74 (16.30)	0.503	0.619	0.089 (–0.259, 0.435)
	VM	72.19 (15.47)	76.64 (15.07)	2.427	**0.021**	0.429 (0.064, 0.788)
	VL	75.42 (17.53)	74.91 (16.69)	0.132	0.896	0.023 (−0.323, 0.370)
	ST	87.65 (17.14)	86.90 (13.48)	0.219	0.828	0.039 (−0.308, 0.385)
	BF	86.81 (14.93)	89.41 (14.75)	0.679	0.502	0.120 (–0.229, 0.467)
	Muscles	Mean (SD)		*t*	*p*	Cohen’s *d* (95% CI)
		MASK	SGS			
MASKvs.SGS	GM	85.76 (17.31)	85.67 (13.47)	0.032	0.974	0.006 (−0.341, 0.352)
	GL	89.14 (19.93)	87.95 (12.49)	0.444	0.66	0.078 (−0.269, 0.425)
	TA	71.25 (20.85)	71.45 (20.24)	0.074	0.942	0.013 (–0.334, 0.359)
	RF	76.74 (13.69)	74.04 (17.19)	1.119	0.272	0.198 (−0.154, 0.546)
	VM	72.19 (15.47)	77.13 (14.27)	1.483	0.148	0.262 (–0.092, 0.613)
	VL	75.42 (17.53)	71.76 (15.28)	1.452	0.157	0.257 (−0.098, 0.607)
	ST	87.65 (17.14)	90.63 (17.33)	1.119	0.272	0.198 (–0.154, 0.546)
	BF	86.81 (14.93)	90.11 (18.71)	1.139	0.264	0.201 (–0.150, 0.550)

Note: GM = Medial Gastrocnemius; GL = Lateral Gastrocnemius; TA = Tibialis Anterior; RF = Rectus Femoris; VM = Vastus Medialis; VL = Vastus Lateralis; ST = Semitendinosus; BF = Biceps Femoris (Long Head). Boldface indicates a significant difference (p < 0.05)

## Discussion

This study investigated whether the targeting effect of the force plate affected participants during running gait data collection under both visible (UNMASK) and masked (MASK) force plate conditions. It also introduced the step guidance strategy (SGS), which aimed to improve the success rate of data collection and reduce or avoid the targeting effect of the force plate. We found that the observed differences in spatiotemporal gait variables, joint kinematics, kinetics, and muscle activity confirmed the presence of the targeting effect of the force plate under the UNMASK condition. However, when data collection was conducted under the proposed SGS condition, this targeting effect could be effectively avoided, and the success rate of data collection was significantly improved.

In our study, the targeting effect resulted in participants experiencing longer gait cycle times and shorter stance phases during running, leading to temporal disturbances (Hirokawa, 1989). No targeting effect was observed in step length, which is inconsistent with previous studies (Abendroth-Smith, 1996; Challis, 2001). This discrepancy may be attributed to differences in the chosen speed or starting distance. Furthermore, our study employed the method of masking the force plate, rendering it visually imperceptible to prevent the deliberate gait modifications associated with targeting effects. In contrast, previous similar studies have merely relied on verbally instructing participants to avoid targeting, conducting visual inspections, or using participant self-reporting to determine whether subjects made conscious adjustments to their stride (Abendroth-Smith, 1996; Challis, 2001). In the coefficient of variation for the distance from the heel to the force plate (HTP CV), we observed results similar to previous studies on the targeting effect in walking (Verniba et al., 2015; Wearing et al., 2000), although the locations of the observed differences were different. In our study, the difference occurred in the second step before the foot made contact with the force plate. In this step, the variability of the HTP distance was significantly reduced under the UNMASK condition, which may suggest that participants adjusted their foot placement to successfully complete the stepping task on the force plate. Previous walking studies observed differences in the step when the foot contacted the force plate and the subsequent step (Verniba et al., 2015), likely due to the faster running speed, which required participants to adjust their steps earlier to complete the stepping task. Another study observed changes in the standard deviation of the HTP distance over more steps (Wearing et al., 2000), however, statistical analysis was not conducted in that study. Under the SGS condition, a similar reduction in the HTP CV was observed, occurring at the step when the foot made contact with the force plate as well as the preceding step. This may be due to the step guidance cues we provided based on each participant's individual step length, making each step more consistent.

The targeting effect of the force plate during running had more pronounced effects on joint angles. Although participants were instructed to avoid directly visualizing the force plate under the UNMASK condition, significant hip flexion was still observed. This may be due to participants subconsciously adopting an aiming posture, such as shifting their center of mass forward, which not only affected the hip joint but also resulted in greater knee flexion during the stance phase and larger plantarflexion and smaller dorsiflexion angles at the ankle during the propulsion phase (Braun et al., 2024). In addition to the changes in the sagittal plane, differences in the hip joint’s coronal and transverse plane movements were also observed. Moreover, the time-domain analysis of GRFs suggested that the targeting effect disrupted the kinetics. A previous study did not observe differences in joint angles or time-domain GRF data (Verniba et al., 2015), while another walking study involving orthopedic patients observed only the effect on hip joint motion in the transverse plane (Bracht-Schweizer et al., 2017). This may suggest that the targeting effect has a greater impact during running than during walking.

Meanwhile, under the UNMASK condition, the variability in ankle joint sagittal plane angle waveforms was greater than under the MASK condition. This increased variability in the ankle joint angle suggests that participants exhibited more variability in ankle movement (O’Dwyer et al., 2009). No significant differences in waveform variability were observed for the three degrees of freedom of the hip joint and the sagittal plane motion of the knee joint, which may indicate that the movement patterns of these two joints are more stable. Additionally, the variability in the anteroposterior GRF waveform was influenced by the targeting effect, while the variability in the vertical and mediolateral directions was not significantly affected. However, previous studies did not report this result (Challis, 2001; Verniba et al., 2015), possibly due to differences in movement patterns (previous studies focused on walking (Grabiner et al., 1995)), and it may also be related to the lack of direct masking of the force plate in those experiments (Challis, 2001; Grabiner et al., 1995). This could also explain why the targeting effect in the present study had a more significant impact on the GRF impulse and peak values, while similar results were not found in prior studies (Challis, 2001; Grabiner et al., 1995; Verniba et al., 2015).

Unlike previous studies, we also examined muscle activity. In Synergy 3 (which represents the propulsion phase) after non-negative matrix factorization, we found significantly higher activation weights for the vastus medialis under the UNMASK condition. Furthermore, higher iEMG values for the vastus medialis were observed under the UNMASK condition. This suggests that under the influence of the targeting effect, the vastus medialis exhibited greater activation, likely due to the larger knee flexion angle under the UNMASK condition, leading to stronger eccentric contraction of the vastus medialis (Green et al., 2018; Grob et al., 2018).

Based on the above discussion, we can make an approximate inference that the presence of visual cues from the force plate leads to the generation of a targeting effect, confirming Hypothesis I. This targeting effect likely causes greater gait disturbances during running than during walking, which would have disastrous consequences for gait analysis, necessitating a reevaluation of prior research (Challis, 2001). Under the MASK condition, although more natural gait data from participants could be collected, the success rate of data collection was only 30%, which is 35.75% lower compared to the UNMASK (65.75%) and 54.21% lower compared to the SGS (84.21%) condition. Furthermore, since we conducted a running experiment, this success rate was also lower than those reported in previous walking studies (Bracht-Schweizer et al., 2017; Rastegarpanah et al., 2018).

This low success rate undoubtedly poses significant challenges for gait data collection in certain special populations, such as individuals with diseases or injuries (Kim and Yu, 2015; Li et al., 2024; Phinyomark et al., 2015), or for conducting specific experiments, such as observing the acute effects of certain interventions (Gao et al., 2023; Janicijevic et al., 2023; Yamane et al., 2025). It also causes psychological distress and physical fatigue for participants after multiple unsuccessful data collections (Chardon et al., 2022; Santos et al., 2019), introduces unnecessary confounding factors into gait analysis, and results in a waste of resources, such as time. Visual access to the force plate improves the success rate of data collection, but due to the targeting effect, the data collected under these conditions may lose authenticity and naturalness (Abendroth-Smith, 1996; Challis, 2001; Hirokawa, 1989). Under the SGS condition, the success rate of data collection significantly increased, surpassing both the MASK and UNMASK conditions. Simultaneously, the targeting effect was effectively reduced. This indicates that the SGS can effectively address both issues, thereby confirming Hypotheses II and III.

Despite these findings, there are still some limitations that should be acknowledged. Firstly, to avoid gait differences caused by speed, we adopted a method to regulate the participants' speed, though it remains unclear whether this measure interfered with their gait. Secondly, the 20-min washout period set in this study remains uncertain in terms of its adequacy. Thirdly, regarding the selection of lower limb joint kinematics, considering the impact of soft tissue artifacts in the placement of reflective markers, three degrees of freedom were selected for the hip joint, while only one degree of freedom in the sagittal plane was selected for both the knee and ankle joints. However, analyzing multiple degrees of freedom remains a viable option. Finally, due to limitations imposed by the experimental environment and running speed, we were unable to collect gait data for a full gait cycle, which may have resulted in the loss of gait information.

## Conclusions

Under conditions where the force plate is visible during running, participants adjust their gait, including modifications in spatiotemporal variables, joint kinematics, kinetics, and muscle activity, resulting in a targeting effect that compromises the reliability of the results. However, the proposed step guidance strategy effectively reduced this targeting effect, while increasing the data collection success rate to 84.21%. We recommend that future research adopts this approach to enhance the success rate of data collection and improve the reliability of research conclusions.
